# Serum fatty acid profiles associated with metabolic risk in women with polycystic ovary syndrome

**DOI:** 10.3389/fendo.2023.1077590

**Published:** 2023-03-31

**Authors:** Ye Tian, Jingjing Zhang, Mingyue Li, Jie Shang, Xiaohong Bai, Huijuan Zhang, Yanxia Wang, Haitao Chen, Xueru Song

**Affiliations:** ^1^ Department of Gynecology and Obstetrics, Tianjin Medical University General Hospital, Tianjin, China; ^2^ Tianjin Key Laboratory of Female Reproductive Health and Eugenics, Tianjin Medical University General Hospital, Tianjin, China; ^3^ School of Public Health (Shenzhen), Sun Yat-sen University, Guangzhou, China; ^4^ School of Public Health (Shenzhen), Sun Yat-sen University, Shenzhen, China

**Keywords:** pcos, fatty acid, metabolic risk, PUFA, GC-MS

## Abstract

**Purpose:**

Dyslipidemia is a feature of polycystic ovary syndrome (PCOS) that may augment metabolic disturbances. Serum fatty acids are important biomedical indicators of dyslipidemia. The aim of this study was to determine the distinct serum fatty acids in various PCOS subtypes and their association with metabolic risk in women with PCOS.

**Methods:**

Fatty acids in the serum of 202 women with PCOS were measured using gas chromatography-mass spectrometry. Fatty acids were compared between PCOS subtypes and correlated with glycemic parameters, adipokines, homocysteine, sex hormones, and sex hormone-binding globulin (SHBG).

**Results:**

The levels of total monounsaturated fatty acids (MUFAs) and polyunsaturated fatty acids (PUFAs) in the reproductive subtype of PCOS were lower than those in the metabolic subtype. Docosahexaenoic acid, a PUFA, was associated with higher SHBG after correction for multiple comparisons. Eighteen species of fatty acids emerged as potential biomarkers associated with the metabolic risk factors measured, independent of body mass index (BMI). Among them, myristic acid (C14:0), palmitoleic acid (C16:1), oleic acid (C18:1n-9C), cis-vaccenic acid (C18:1n-7), and homo-gamma-linolenic acid (C20:3n-6) were the strongest lipid species that were consistently associated with metabolic risk factors, particularly insulin-related parameters in women with PCOS. As for adipokines, 16 fatty acids were positively associated with serum leptin. Among them, C16:1 and C20:3n-6were significantly associated with leptin levels.

**Conclusion:**

Our data demonstrated that a distinct fatty acid profile comprising high C14:0, C16:1, C18:1n-9C, C18:1n-7, and C20:3n-6levels is associated with metabolic risk in women with PCOS, independent of BMI.

## 1 Introduction

Polycystic ovary syndrome (PCOS) is a complex disorder affecting 5%–20% of women of reproductive age worldwide (1). It is characterized by hyperandrogenism (HA; hirsutism, persistent acne, and/or biochemical hyperandrogenemia), ovulatory dysfunction (oligo/anovulation, menstrual irregularity, and infertility), and polycystic ovarian morphology (an excessive number of preantral follicles in the ovaries).PCOS is associated with metabolic abnormalities and possibly with an increased risk of metabolic syndrome, type II diabetes mellitus, and cardiovascular diseases (2).

Dyslipidemia is an increasingly common feature, reported in 41%–70% of women with PCOS (3–5). Increased triglyceride and low-density lipoprotein cholesterol levels and decreased high-density lipoprotein cholesterol(HDL-C) levels are common lipid abnormalities in women with PCOS (6). Dyslipidemia plays an important role in metabolic and endocrine pathways in women with PCOS. Drugs used to reduce the level of cholesterol in PCOS cases could cause a reduction in the levels of total testosterone, free androgen index(FAI)and dehydroepiandrosterone sulfate; however, the certainty of the evidence is low (7).Beyond known risk factors for cardiovascular disease, decreased HDL-C levels and elevated triglyceride levels are also reportedly associated with a lower maturation rate in women with PCOS undergoing *in vitro* maturation (8).

Apart from conventional lipid profiling, including for triglycerides, total cholesterol, low-density lipoprotein cholesterol, and HDL-C, free fatty acids are also important biomedical indicators of dyslipidemia. Fatty acids are efficient substrates for energy production and may directly affect the metabolism of various lipid and glycemic molecules (9, 10). However, the association between fatty acid profiles and metabolic risk in PCOS cases has not been comprehensively explored (11).

The signs and symptoms of PCOS are heterogeneous and can be categorized into several subtypes. Dyslipidemia may differ among subsets of women with PCOS. Lower HDL-C levels may also be related to hyperandrogenemia in lean PCOS patients (12). However, the fatty acid levels in PCOS patients with or without hyperandrogenemia have not been well defined. In addition, Dapas et al. recently performed a clustering analysis in women with PCOS and identified two distinct subtypes: reproductive and metabolic (13). It remains unclear whether these two subtypes have distinct fatty acid profiles.

As a branch of targeted metabolomics, absolute quantification of fatty acids is used to systematically investigate many fatty acids rather than focusing on a single specific fatty acid. In this study, we carried out systematic fatty acid profiling of PCOS cases of various subtypes using gas chromatography-mass spectrometry. We aimed to investigate the distinct fatty acid profiles of different PCOS subtypes and define the associations between fatty acids and metabolic risk factors.

## 2 Methods

### 2.1 Patients

This cohort consisted of 202 Han Chinese women with PCOS and 34 women without PCOS, recruited from the Reproductive Center, Tianjin Medical University General Hospital. PCOS was defined using the 2003 Rotterdam PCOS consensus criteria (14), and other related diseases with similar presentations (congenital adrenal hyperplasia, androgen-secreting tumors, Cushing’s syndrome, thyroid disease, and hyperprolactinemia) were excluded. PCOS diagnosis requires two of the following criteria: oligo/anovulation (menstrual cycle length >35 days), clinical or biochemical HA(Ferriman-Gallwey score ≥6, severe acne, or total testosterone≥57ng/dL), and polycystic ovarian morphology (at least 12 follicles measuring 2–9 mm in diameter in one ovary and/or increased ovarian volume >10 mL on ultrasound). The inclusion criteria for the control group were as follows: normal menstrual cycles, and neither HA nor polycystic ovaries under ultrasound. Individuals taking medications, such as oral contraceptives and metformin, during the previous 3 months were also excluded. The study was approved by the Ethics Committee of Tianjin Medical University General Hospital, and written informed consent was obtained from all the participants.

### 2.2 Clinical and biochemical measurement

All participants were assessed for age, height, and weight. Body mass index (BMI) was calculated as weight (kg)/height (m)^2^. Fasting blood samples were obtained on days 2–4 of the menstrual cycle to examine circulating serum levels of hormones, including follicle-stimulating hormone (FSH), luteinizing hormone (LH), and total testosterone. Serum sex hormone-binding globulin (SHBG)and dehydroepiandrosterone sulfate were measured using a chemiluminescence immunoassay (IMMULITE1000, USA). Insulin levels were also measured using a chemiluminescence immunoassay (ARCHITECT i2000SR, USA), and fasting glucose (Glu0) was measured using the glucose oxidase method (VITROS 5600, USA).The FAI was calculated as follows: testosterone (nmol/L)/SHBG (nmol/L) × 100. The homeostasis model assessment for insulin resistance (HOMA-IR) was calculated as Glu0 (mmol/L) × fasting insulin (mIU/L)/22.5.

### 2.3 Enzyme-linked immunosorbent assay

Adiponectin and leptin concentrations were determined using an enzyme-linked immunosorbent assay with specific commercial kits designed for humans (CUSABIO, China). All procedures were performed according to the manufacturer’s instructions.

### 2.4 Metabolite extraction

The serum was pipetted into a 15-mLcentrifuge tube, and 2 mL of 1% sulfuric acid methanol solution was added. Thereafter, esterification was performed in an 80°C water bath for 30 min.After the mixture was removed from the water and cooled,1 mL of n-hexane was added for extraction.The mixture was shaken and mixedfor 30 sand incubated upright at room temperaturefor 5 min.Subsequently,5 mL of H_2_O (4°C) was added for washing, and the mixture was centrifuged at 3500 rpm at 4°C for 10 min.The supernatant (700 μL) was carefully transferred to a 2-ml centrifuge tube, and 100 mg of anhydrous sodium sulfate powder was added to remove excess water. The mixture was shaken and mixed for 30 s and centrifuged at 12000 rpm for 5 min. The supernatant (200 μL) was carefully transferred to a 2-mL centrifuge tube, and 200 mL of n-hexane was added. Further, 300μL of the diluent was carefully transferred to a 2-mL centrifuge tube and 15 ml of 500 ppm methyl salicylate was added as an internal standard. The mixture was shaken and mixed for 10s, and 200 μL supernatant was accurately absorbed in the detection bottle.

### 2.5 Gas chromatography-mass spectrometry method

A gas chromatograph coupled to a mass spectrometry system (Thermo Trace 1310 GC-Thermo ISQ 7000, Thermo-Fisher Scientific Corp., FairLawn, NJ, USA) was used to quantify fatty acids at Novogene Co., Ltd. (Beijing, China). The system utilized a Thermo TG-Fame capillary column (50 m×0.25 mm, internal diameter = 0.20 μm). A 1 μL aliquot of the sample was injected into the system. Helium was used as the carrier gas, and the gas flow rate through the column was 0.63 mL/min. The initial temperature was maintained at 80°C for 1min, raised to 160°C at a rate of 20°C/min, and maintained for 1.5 min. Thereafter, it was increased to 196°C at a rate of 3°C/min and maintained for 8.5 min. Finally, it was raised to 250°C at a rate of 20°C/min and maintained for 1.5 min. The mass spectrometer was operated in the single-ion monitoring mode. The energy in the electron impact ion source was 70 eV.

### 2.6 Clustering

Clustering was performed for 202 PCOS cases on eight quantitative traits: BMI, total testosterone, dehydroepiandrosterone sulfate, fasting insulin (Ins0), Glu0, SHBG, LH, and FSH, according to a previous publication (13). Briefly, quantitative trait values were normalized and clustered using unsupervised, agglomerative, and hierarchical clustering according to Ward’s minimum variance method on Manhattan distances between trait values. The Complex Heatmap software package was used to visualize the distributions of clustering of PCOS cases with different quantitative trait levels.

### 2.7 Statistical analysis

Quantitative variables of clinical characteristics of PCOS subjects are displayed as the mean ± standard error. Values for each fatty acid were log_10_ transformed because most had a naturally skewed distribution. Fatty acid classes, including saturated fatty acids (SFAs), monounsaturated fatty acids (MUFAs), polyunsaturated fatty acids (PUFAs), and trans fatty acids (TFAs), were generated by summing the individual fatty acid concentrations that make up each class. Statistical analysis was performed using IBM SPSS version22.0 for Windows (IBM Corp., Armonk, NY, USA) or R software (4.0.5). Normality was assessed using the Kolmogorov–Smirnov test. Differences between the PCOS and control groups or two PCOS subgroups were assessed using individual sample t-tests for normal distributions or Mann–Whitney U tests for non-normal distributions. Univariate and multivariable linear regressions were used to determine associations between fatty acids and the outcomes of interest, and age and BMI were used as covariates in the analysis. We corrected for the false discovery rate (FDR) by using the Benjamini–Hochberg method, and statistical significance was set at *P*<0.05.

## 3 Results

### 3.1 Characteristics of the patients

The anthropometric, clinical, and biochemical characteristics of the 202 PCOS patients are shown in **Table 1**. The mean age of the PCOS subjects was 30.78years, and the mean BMI was 25.08 kg/m^2^. For PCOS-related endocrine parameters, the cases had a significantly higher total testosterone level, LH level, FAI, and anti-Müllerian hormone level(all *P*<0.01) than controls. Except for higher leptin levels in women with PCOS, there were no differences in other metabolic characteristics between BMI-matched women with and without PCOS, including fasting blood glucose, fasting serum insulin, HOMA-IR, and adiponectin levels (all *P*>0.05). Fatty acid components significantly differed between women with and without PCOS after adjusting for age. The levels of total MUFAs and PUFAs in the PCOS group were markedly lower, whereas those of SFAs and TFAs were significantly higher in the PCOS group than those in the control group (**Table 1**).

**Table 1:** Characteristics of PCOS cases and controls.

	PCOS (n=202)	Controls (n=34)	*P*
Age (years)	30.78 ± 3.66	31.94 ± 3.56	0.042
BMI (kg/m^2^)	25.08 ± 4.66	23.73 ± 4.26	0.117
Follicle-stimulating hormone (IU/L)	5.22 ± 1.45	6.14 ± 2.29	0.055
Luteinizing hormone (IU/L)	7.12 ± 4.49	3.97 ± 2.16	<0.001
Total testosterone (ng/dL)	46.86 ± 17.95	24.18 ± 8.03	<0.001
Sex hormone-binding globulin(nmol/L)	57.46 ± 60.56	44.67 ± 28.89	0.811
Free androgen index	5.40 ± 4.73	2.69 ± 1.98	0.002
Anti-Müllerian hormone (ng/mL)	8.25 ± 5.34	2.13 ± 0.87	<0.001
Fasting glucose (mmol/L)	5.26 ± 1.34	4.94 ± 0.59	0.086
Fasting insulin(mIU/L)	13.69 ± 8.82	12.04 ± 7.27	0.327
HOMA-IR	3.32 ± 2.64	2.73 ± 1.89	0.187
Leptin (ng/mL)	34.88 ± 14.06	22.41 ± 14.57	<0.001
Adiponectin (μg/mL)	33.79 ± 15.40	38.96 ± 13.53	0.082
Log_10_total SFAs (μg/mL)	3.17 ± 0.10	3.06 ± 0.12	<0.001
Log_10_total MUFAs (μg/mL)	2.48 ± 0.18	2.68 ± 0.17	<0.001
Log_10_total TFAs (μg/mL)	1.77 ± 0.26	1.70 ± 0.13	0.008
Log_10_total PUFAs (μg/mL)	2.57 ± 0.16	2.71 ± 0.19	<0.001

PCOS, polycystic ovary syndrome; BMI, body mass index; HOMA-IR, homeostasis model assessment for insulin resistance; SFAs, saturated fatty acids; MUFAs, monounsaturated fatty acids; PUFAs, polyunsaturated fatty acids; TFAs, trans fatty acids.

### 3.2 Serum fatty acids in PCOS subtypes

**Figure 1:**
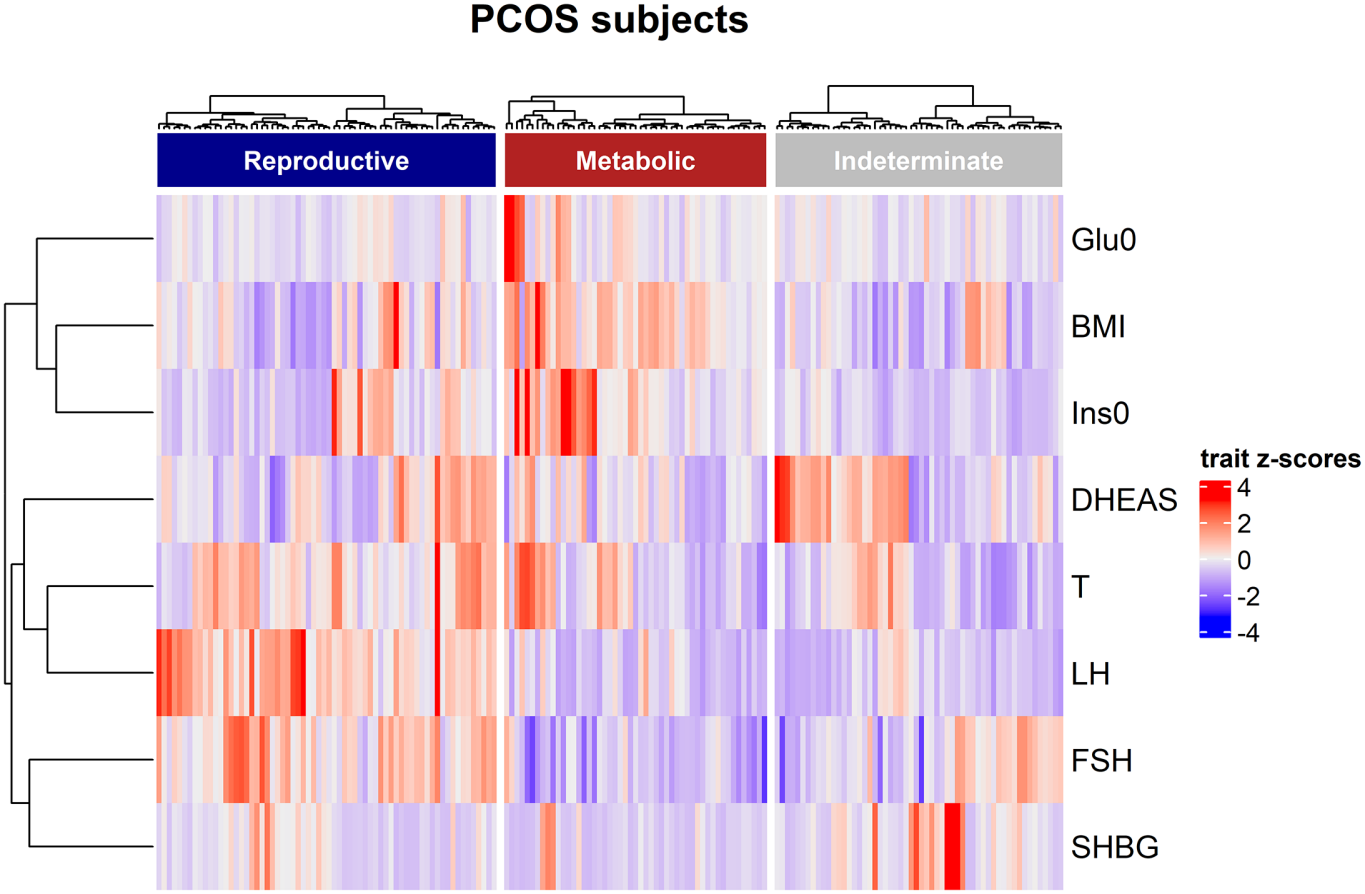
Hierarchical clustering of genotyped PCOS clustering cohort. The three clusters are shown as dark blue, dark red, and grey color bars. Heatmap colors correspond with the trait Z-scores, as shown to the right side, in which red implies high values and blue indicates low values for each trait. The trait-based dendrogram represents relative distances between the trait distributions and was calculated using the same approach as that for the subject-based clustering.

In women with PCOS with or without the HA subtype, the levels of serum fatty acids were similar after adjusting for age and BMI (**Table 2**). Clustering was performed for the 202 PCOS cases (**Figure 1**). Clustering revealed two distinct phenotypic subtypes: the reproductive subtype (n=55, higher LH and SHBG levels with relatively low BMI and Ins0 levels) and the metabolic subtype (n=65, higher BMI and Glu0 and Ins0 levels with relatively low SHBG and LH levels).The remaining cases showed no distinguishable patterns after clustering. The levels of total MUFAs and PUFAs in the reproductive subtype of PCOS were lower than those in the metabolic subtype (**Table 2**).

**Table 2:** Distributions of serum fatty acids in various PCOS subtypes.

	HA subtype (n=50)	Non-HA subtype (n=144)	*P*	*P*-adj^a^	Reproductive subtypes (n=55)	Metabolic subtypes (n=65)	*P*	*P*-adj^b^
Age (years)	30.32 ± 4.2	30.89 ± 3.4	0.343	–	30.65 ± 3.5	30.42 ± 3.9	0.726	–
BMI (kg/m^2^)	26.24 ± 5.7	24.69 ± 4.2	0.043	–	23.46 ± 3.7	24.35 ± 4.2	0.277	–
Log_10_total SFAs (μg/mL)	3.19 ± 0.11	3.16 ± 0.10	0.074	0.087	3.16 ± 0.10	3.17 ± 0.11	0.713	0.715
Log_10_total MUFAs (μg/mL)	2.51 ± 0.18	2.47 ± 0.17	0.099	0.154	2.42 ± 0.14	2.49 ± 0.15	0.016	0.018
Log_10_total TFAs (μg/mL)	1.78 ± 0.25	1.78 ± 0.30	0.997	0.924	1.74 ± 0.23	1.79 ± 0.21	0.282	0.287
Log_10_total PUFAs (μg/mL)	2.60 ± 0.15	2.56 ± 0.16	0.143	0.166	2.52 ± 0.15	2.59 ± 0.15	0.009	0.012
Log_10_n-3 PUFA (μg/mL)	1.61 ± 0.15	1.57 ± 0.17	0.152	0.139	1.54 ± 0.16	1.60 ± 0.16	0.027	0.029
Log_10_n-6 PUFA (μg/mL)	2.51 ± 0.16	2.55 ± 0.15	0.146	0.174	2.47 ± 0.16	2.55 ± 0.15	0.009	0.012

PCOS, polycystic ovary syndrome; HA, hyperandrogenism; P-adj^a^, Padjusted by age and BMI; P-adj^b^, Padjusted by age; SFAs, saturated fatty acids; MUFAs, monounsaturated fatty acids; PUFAs, polyunsaturated fatty acids; TFAs, trans fatty acids.

### 3.3 Associations between serum fatty acid and sex hormone concentrations

The associations of fatty acids with FSH, LH, total testosterone, FAI, SHBG, and anti-Müllerian hormone are shown in **Supplementary Table 1**. Docosahexaenoic acid (DHA, C22:6N3), which belongs to the PUFA class, was associated with higher SHBG levels after FDR correction (*P*<0.05). Mild correlations were observed between some fatty acids and FSH or FAI; however, none of these correlations were significant after correction for multiple comparisons. In addition, no significant associations were detected between a single fatty acid and LH, total testosterone, or anti-Müllerian hormone levels.

### 3.4 Associations between serum fatty acids and metabolic parameters

**Figure 2:**
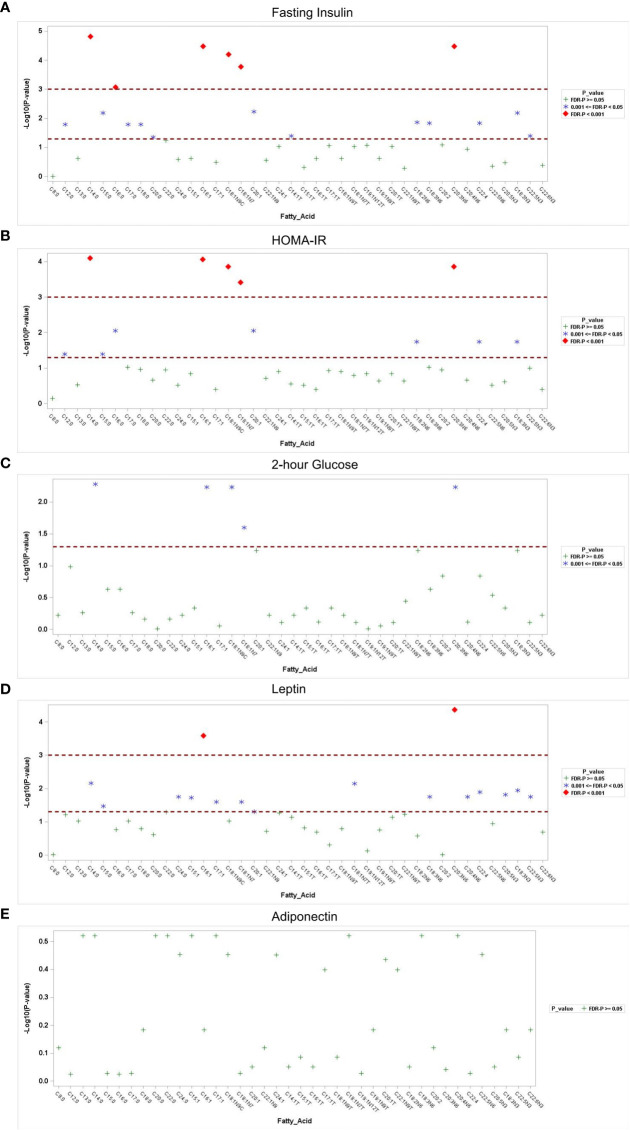
Association of fatty acids with metabolic parameters. Manhattan plots of the -log_10_p-value for the fasting insulin **(A)**, HOMA-IR **(B)**, 2-hour glucose **(C)**, serum leptin **(D)** and adiponectin **(E)** regressed on log-transformed fatty acids. Each y-axis represents the-log_10_p-value for each respective model, and each x-axis represents the serum fatty acid. Serumfatty acids with Benjamini–Hochberg false discovery rate-corrected *p*-values <0.05 and *p*-values <0.001 are separated.

Associations of fatty acids with Glu0, 2-h glucose,Ins0, HOMA-IR, leptin, adiponectin, and homocysteine are shown in **Supplementary Table 2** and **Figure 2**. No significant associations were observed with Glu0, but 2-hour glucose after the 75-g oral glucose tolerance test was positively associated with myristic acid (C14:0),palmitoleic acid (C16:1),oleic acid (C18:1n-9C),cis-vaccenic acid (C18:1n-7), and homo-gamma-linolenic acid (C20:3n-6), all of which was significant after FDR correction(*P*<0.05).After adjustment for age and BMI, the associations between the five types of fatty acid and 2-hour glucose were still significant. In total, 18 fatty acid species were significantly associated with Ins0 levels (FDR-corrected *P*<0.05). Most notably, six fatty acids, that is, myristic acid (C14:0), hexadecanoic acid (C16:0), palmitoleic acid(C16:1),oleic acid (C18:1n-9C), cis-vaccenic acid(C18:1n-7), and homo-gamma-linolenic acid (C20:3n-6),emerged as the lipid biomarkers most strongly associated with Ins0 (FDR-corrected *P*<0.001).Consistently, 12 species of fatty acids were significantly associated with HOMA-IR; the strongest associations were with myristic acid (C14:0), palmitoleic acid(C16:1), oleic acid (C18:1n-9C), cis-vaccenic acid(C18:1n-7), and homo-gamma-linolenic acid(C20:3n-6)after adjustment for age and BMI (FDR-corrected *P*<0.001).

As for adipokines, 16 fatty acids were positively associated with serum leptin using multiple longitudinal models, even after adjustment for age (FDR-corrected *P*<0.05). Among them, palmitoleic acid (C16:1) and homo-gamma-linolenic acid (C20:3n-6) were most significantly associated with the level of leptin (FDR-corrected *P*<0.001). However, no significant correlations were detected between fatty acids and adiponectin or homocysteine levels after correction for multiple comparisons (FDR-corrected *P*>0.05).

## 4 Discussion

In the present study, we performed absolute quantification of 51 fatty acids in the serum and found that the reproductive subtype of PCOS cases has a distinct fatty acid profile compared to the metabolic subtype. Specific fatty acids, including myristic acid (C14:0),palmitoleic acid (C16:1),oleic acid (C18:1n-9C),cis-vaccenic acid (C18:1n-7), and homo-gamma-linolenic acid (C20:3n-6) were associated with metabolic risk factors, including 2-hour glucose, Ins0, and HOMA-IR, in PCOS patients, independent of BMI.

In a previous study, lipidomic analysis of plasma samples identified differences in lipidomic profiles in women with PCOS at different stages of the menstrual cycle (15); however, the results were inconsistent with those of other studies (16, 17), partly owing to the recruited PCOS populations and characteristics. In this study, we found that the levels of total MUFAs and PUFAs in the PCOS group were lower than those in the control group, whereas the levels of SFAs and TFAs were higher in the PCOS group. Consistent with our study, Li et al. and Lu et al. found that PCOS patients had decreased PUFA levels and increased levels of SFAs in serum compared to lean controls (18, 19). In the subcutaneous adipose tissue of pregnant women with PCOS, the level of total PUFAs was lower, while that of total MUFAs was higher than that in non-PCOS women.

The lipid profile was significantly more disordered in the full-blown PCOS group (oligo/anovulation+HA+ polycystic ovaries) with higher low-density lipoprotein cholesterol and total cholesterol and lower HDL values than the non-HA PCOS group (oligo/anovulation+polycystic ovaries) (20). However, the fatty acid profiles of women with HA and non-HA PCOS remain unclear. In the present study, no significant differences in the levels of SFAs, TFAs, MUFAs, or PUFAs were found between women with PCOS with and without HA after adjustment for age and BMI. Recently, two distinct subtypes (reproductive and metabolic) have been identified in women with PCOS (13). We found that the total MUFAs and PUFAs in the reproductive subtype of PCOS cases were even lower than those in the metabolic subtype, indicating that a unique fatty acid profile was associated with each of the PCOS subtypes. The subtypes of PCOS were generated by cluster analysis of eight quantitative hormonal and glycemic parameters, and it is possible that the addition of lipid parameters may optimize clustering.

Lower SHBG levels are usually used as an indicator of HA in women with PCOS, considering its binding to androgens. In addition, a low serum SHBG level is related to insulin resistance (IR) and, hence, is considered an indicator of abnormal metabolism (21). Here, we report a positive association between SHBG and DHA in women with PCOS. However, other fatty acids were not significantly associated with SHBG after correction for multiple comparisons. Consistent with our study, Mousa et al. found that lower DHA concentrations were associated with lower SHBG and higher adiposity, IR, Ins0, and FAI (17). Lu et al. found that serum DHA levels were positively correlated with FSH and SHBG but negatively correlated with Ins0andtotal testosterone (19). In addition, DHA is one of the most well-known omega-3 PUFAs (n-3 PUFAs), and several studies have evaluated the changes in hormones in women with PCOS after supplementation with n-3PUFA; however, the results have been mixed. Increased dietary PUFA intake can exert positive metabolic and endocrine effects in women with PCOS; however, there is no change in SHBG level (22). Conversely, according to a meta-analysis, PCOS patients with n-3 PUFA supplementation had an increase in serum SHBG (0.68 mg/dL; 95% confidence interval:0.06 to 1.31 mg/dL) (23). The functional mechanism by which DHA regulates SHBG levels is unclear. PUFAs are ligands of peroxisome proliferator-activated receptors (PPARs), and the human SHBG promoter contains a PPAR-response element. Thus, DHA may regulate SHBG levels through PPARs (24). Importantly, administration of the PPAR agonist, rosiglitazone, to women with PCOS increases their serum SHBG levels (25).

IR is a key pathophysiological feature of PCOS and is likely to contribute to dyslipidemia. Lipolysis mainly occurs in the adipose tissue and an enlarged adipose tissue mass can release more free fatty acids. In patients with IR, the rate of lipolysis is higher, leading to an increase in fatty acid delivery to the liver and muscle, and the oxidation of fatty acids is compromised (26). Increased levels of free fatty acids have deleterious effects and further contribute to IR, considering that ectopic triglyceride accumulation in the liver and skeletal muscle triggers pathways that impair insulin signaling (27). The underlying mechanisms of free fatty acid-induced IR are as follows: activation of serine/threonine kinases, reduced tyrosine phosphorylation of the insulin receptor substrate (IRS1/2), and impairment of the IRS/PI3K pathway (28). Free fatty acids can lead to the generation of reactive oxygen species and act as modulators of the NLRP3 inflammasome, which may play an important role in IR (29, 30). In our study, the serum fatty acid profiles indicated dramatically increased levels of 12 fatty acids in PCOS patients in the IR group compared to those in the non-IR group, irrespective of obesity. Most notably, five fatty acids were strongly related to IR (FDR-corrected *P*<0.001), including myristic acid, palmitoleic acid, oleic acid, cis-vaccenic acid, and homo-gamma-linolenic acid. Moreover, we also found that these five fatty acids were significantly correlated with 2-hour glucose and Ins0 levels, which indicated that they were potential lipid biomarkers for metabolic risk in PCOS patients. Our findings are similar to those of Holte et al., who found that the concentrations of total free fatty acids were closely associated with lower insulin sensitivity and lower glucose tolerance in women with PCOS (31); however, they only focused on the total rather than each specific fatty acid. Niu et al. also studied the total free fatty acid content and found a weak correlation between total fatty acid levels in follicular fluid and IR in lean PCOS patients (32).

Previous studies have shown the levels of myristic acid, palmitoleic acid, and oleic acid to be higher in women with PCOS, with conflicting results (33–35). In another study, *de novo* fatty acid synthesis of myristic acid, palmitoleic acid, and oleic acid in subcutaneous adipose stem cells of normal-weight PCOS patients was increased compared to that in controls (36). According to a serum metabolomics study of 20 women with PCOS, myristic acid and palmitoleic acid levels were significantly higher in IR PCOS patients than in non-IR PCOS patients (33). In contrast, another research group reported that palmitoleic acid and oleic acid, but not myristic acid, were strongly associated with IR in girls with PCOS (37). It is unclear why studies on women with PCOS have yielded conflicting results, but this may be due, in part, to the small number of PCOS cases in the studies. In addition, differences in patient characteristics and lipid extraction and analysis methods were observed. To our knowledge, few studies have explored the role of cis-vaccenic acid and homo-gamma-linolenic acid in PCOS (38). Our data, for the first time, showed that both cis-vaccenic acid and homo-gamma-linolenic acid were associated with IR in a relatively large cohort of PCOS patients, emphasizing the need for further studies to clearly define the role of these two fatty acids in PCOS and determine the functional mechanism underlying the actions of insulin.

Leptin and adiponectin, which are adipokines secreted by adipocytes, have a profound influence on insulin sensitivity (39). Women with PCOS present with adipokine alterations, such as increased leptin levels and decreased adiponectin levels. Fatty acid concentration is an important factor that can influence adipokines (40). PCOS patients with a higher n-3 PUFA intake have may have increased serum adiponectin and reduced leptin concentrations; however, the results have been mixed (41). In this study, 16 fatty acids were positively associated with serum leptin, the most significant of which were palmitoleic acid and homo-gamma-linolenic acid. We speculate that these two fatty acids play an important role in IR through leptin-related mechanisms. Previously, homo-gamma-linolenic acid was found to be positively associated with plasma leptin throughout pregnancy in a prospective study of 201 pregnant women (42). The mechanism by which these two fatty acids might affect the level of leptin is unclear, and thus, further study is needed to understand how they contribute to the biology of PCOS.

## 5 Conclusion

In summary, we report that two distinct reproductive and metabolic subtypes of women with PCOS have unique fatty acid profiles. Our data demonstrated thata distinct fatty acid signature comprising high levels of myristic acid (C14:0),palmitoleic acid (C16:1),oleic acid (C18:1n-9C),cis-vaccenic acid (C18:1n-7), and homo-gamma-linolenic acid (C20:3n-6) is particularly associated with insulin-related metabolic risk in women with PCOS, independent of BMI. Additionally, DHA was positively associated with SHBG levels. Although the underlying mechanisms are not fully understood, our findings provide new evidence supporting the important role of these fatty acids in PCOS. Further studies are needed to verify these findings in larger cohorts of PCOS patients.

## Data availability statement

The original contributions presented in the study are included in the article/**Supplementary Material**. Further inquiries can be directed to the corresponding authors.

## Ethics statement

The studies involving human participants were reviewed and approved by Institutional Review Board of Tianjin Medical University General Hospital. The patients/participants provided their written informed consent to participate in this study.

## Author contributions

YT and XS designed and provided support during the study. ML, HZ, YW, and XS collected all the clinical data and blood samples. ML and JS performed the experiments. YT, JZ, and HC analyzed the data. YT drafted the manuscript. XB and XS revised the article. All authors contributed to the article and approved the submitted version.
